# Psychological distress among healthcare providers in oncology during the COVID-19 pandemic in Japan: The mediating role of moral distress and resilience

**DOI:** 10.3389/fpsyg.2023.1105800

**Published:** 2023-02-01

**Authors:** Masako Okamura, Maiko Fujimori, Shinichi Goto, Keiko Ohisa, Narikazu Boku, Rika Nakahara, Yosuke Uchitomi, Tatsuya Suzuki, Tomohiro Matsuda

**Affiliations:** ^1^Division of Supportive Care, Survivorship and Translational Research, National Cancer Center Institute for Cancer Control, Tokyo, Japan; ^2^Department of Gastrointestinal Medical Oncology, National Cancer Center Hospital, Tokyo, Japan; ^3^Department of Psycho-Oncology, National Cancer Center Hospital, Tokyo, Japan; ^4^Department of Hematology, National Cancer Center Hospital, Tokyo, Japan; ^5^Strategic Planning Bureau, National Cancer Center, Tokyo, Japan; ^6^Division of International Health Policy Research, National Cancer Center Institute for Cancer Control, Tokyo, Japan

**Keywords:** COVID-19, health personnel (MeSH), moral distress, psychological distress, resilience, cancer care

## Abstract

**Objective:**

Even though vaccines have become widespread, there is an explosion of infection due to the emergence of new mutant strains, and support for healthcare providers’ mental health is necessary. The aims of this study were to explore factors associated with the psychological distress, and to determine the degree of association between moral distress, resilience and psychological distress in order to consider intervention models for psychological distress of healthcare providers involved with cancer patients during the COVID-19 pandemic.

**Method:**

We conducted a cross-sectional survey among healthcare providers at the National Cancer Center, Japan. Psychological distress was assessed by the Hospital Anxiety and Depression Scale. We also assessed moral distress using the Moral Distress Thermometer and resilience using the Connor-Davidson Resilience Scale 10 in April and May 2020 which was the first surge of the epidemic period.

**Results:**

Five hundred sixty-six of 3,900 healthcare providers (14.5%) responded. Sixty-eight percent (385/566) responders were above the Hospital Anxiety and Depression Scale cutoff. Hierarchical regression analyses indicated that nurses, allied health professionals and office workers/engineers (odds ratio = 4.63; 95% confidence interval 1.90–11.29; *p* < 0.001, odds ratio = 3.88; 95% confidence interval 1.88–8.00; *p* < 0.001, odds ratio = 2.10; 95% confidence interval 1.06–4.18; *p* < 0.05) as well as healthcare providers with low resilience (odds ratio = 0.88; 95% confidence interval 0.85–0.91; *p* < 0.001) were at risk of psychological distress. Moral distress was not significantly associated with prevalence of psychological distress.

**Conclusion:**

During the first surge of the pandemic, a high prevalence of psychological distress was demonstrated among cancer center healthcare providers. The study suggests that mental health care should be available to cancer care providers. Since the COVID-19 pandemic is still going on, in addition to the efforts by our facilities, we should consider interventions that promote resilience and a feasibility study of these interventions.

## Introduction

The coronavirus disease 2019 (COVID-19) has seriously threatened human health and has affected many aspects of life. Studies show the significant impact of the outbreak on mental health (Vindegaard and Benros, 2020; Wu et al., 2021). Healthcare providers (HCP) are in the most vulnerable group at risk of mental health problems. Systematic reviews regarding mental health among HCPs reported that they had moderate to high levels of psychological distress such as depression, anxiety, insomnia, and acute and posttraumatic stress symptoms (Carmassi et al., 2020; Papa et al., 2020; Maqbali et al., 2021).

HCPs caring for cancer patients face some unique challenges. In addition to the stressors associated with caring for patients with cancer, HCPs currently have pandemic-related concerns (Bakouny et al., 2020; Schrag et al., 2020). There is a risk that immunocompromised patients by treatment would be infected with the COVID-19, and clusters might occur in hospitals. Therefore, cancer treatment and care must be carried with great care and are accompanied by a higher degree of tension.

HCPs in oncology might be prone to moral distress when deciding between cancer care and infection control. Moral distress is defined as the discomfort felt when a person, institution, or situation prevents a HCP from doing what he or she believe is morally right in health care (Sonis et al., 2022). Studies of moral distress among HCPs related to the COVID-19 pandemic have shown associations between moral distress and mental health issues, including anxiety, depression and post-traumatic stress disorder (PTSD; Norman et al., 2021; Petrisor et al., 2021; Schneider et al., 2021; Lake et al., 2022). On the other hand, there are research reports on factors that reduce the outcome of psychological distress after adverse experiences. These are known as resilience factors. Resilience factors include a strong sense of purpose, adaptive and coping capacity, positive mental state, self-confidence, optimism and perceived strong social support (Kunzler et al., 2020; Hines et al., 2021). Resilience is an innate physical and psychological trait that an individual possesses (Jackson et al., 2007; Fox et al., 2018). Psychological resilience is the ability to positively adapt to life conditions. It is a dynamic process that evolves over time, implying adaptive capabilities that allows us to face challenges by restoring initial balance or bouncing back as opportunities for growth (Sisto et al., 2019). The resilience protective model (Bonanno, 2004) shows that interactions between protective factors and risks can promote positive health outcomes despite adverse or aversive situations (Laura et al., 2021).

In Japan, we were in the middle of the seventh surge of the pandemic in August 2022 and had the highest infection rate ever. The Japanese government declared the first state of emergency in April and May 2020. Since then, there have been reports of deterioration in the mental health of medical personnel (Sasaki et al., 2020; Tahara et al., 2021). Even though vaccines have become widespread, there is an explosion of infection due to the emergence of new mutant strains, and support for HCPs’ mental health is necessary. Therefore, the aims of this study were to explore factors associated with the psychological distress, and to determine the degree of association between moral distress, resilience and psychological distress in order to consider intervention models for psychological distress of HCPs involved with cancer patients during the COVID-19 pandemic. We hypothesized that HCPs with high moral distress would have psychological distress and resilience would be a protective predisposition to psychological distress.

## Materials and methods

### Participants and procedure

This study is part of the international project with two other oncology hospitals in United Kingdom and Canada. We conducted a cross-sectional survey among healthcare workers (doctors, nurses, allied health professionals, researchers, and office workers) in the two hospitals of National Cancer Center (NCC), Japan. The NCC Hospital (NCCH) is in Tokyo with 578 beds and NCC Hospital East (NCCHE) is in Chiba with 425 beds. Both facilities have between 1,200 and 1,500 outpatients per day. Due to the spread of COVID-19 infection, NCCH has been accepting hospitalization of COVID-19 infected patients in one ward with 25 beds at the request of the Tokyo Metropolitan Government since 15 April 2020, even if they do not have cancer. This survey was conducted between 7 July and 27 July 2020. Participants were asked their psychological status in April and May, which was the first surge of the epidemic period. An informed consent letter was emailed to participants, and completion of the questionnaire implied their consent. This study was approved by the Institutional Review Board and Ethics Committee of NCC, Japan, and was carried out in accordance with the principles set out in the Helsinki Declaration.

### Measures

#### Psychological distress: The hospital anxiety and depression scale

The hospital anxiety and depression scale (HADS) is a self-reported questionnaire evaluating depression and anxiety with 7 items each (Zigmond and Snaith, 1983). Each item is rated on a four-point Likert scale of 0, 1, 2, and 3. Higher scores indicate severe symptoms of anxiety and depression. The validity and reliability of the Japanese version of the HADS have been established (Kugaya et al., 1998). People with scores of 11 or more are classified as having significant psychological distress. The Cronbach’s α coefficients of the HADS was 0.77 in this survey.

#### Moral distress: The moral distress thermometer

The Moral Distress Thermometer is a one-item visual-analog scale for rating moral distress. After receiving a short explanation of moral distress, participants rated the amount of moral distress that they had experienced in the previous 2 weeks, rated from 0 to 10 (Wocial and Weaver, 2013). It was designed as a screening tool targeted toward nurses in a hospital setting and has good convergent and discriminant validity (Wocial and Weaver, 2013). It has been used to measure moral distress in HCPs (Mehlis et al., 2018; Sonis et al., 2022). The Japanese translation of the Moral Distress Thermometer was handled by a native English-speaking researcher who is fluent in Japanese and a Japanese-speaking researcher who is fluent in English.

#### Resilience: The Connor-Davidson resilience scale 10

The Connor-Davidson resilience scale (CD-RISC) was developed with the aim to include measures of resilience in managing individuals with PTSD and other anxiety forms. The CD-RISC 10 evaluates resilience using 10 items and scoring of this scale is based on the sum of scores from 0 (not true at all) to 4 (true nearly all the time) for each item (range: 0–40). A higher score indicates a greater level of resilience (Connor and Davidson, 2003). This scale has been studied and applied across diverse populations, including physicians and office workers (Awano et al., 2020). The reliability and validity of the Japanese version of the CD-RISC has been confirmed (Nishi et al., 2010). The Cronbach’s α coefficients of the CD-RISC was 0.92 in this survey.

#### Sociodemographic characteristics:

Sociodemographic information was obtained from the participants (Table 1).

**Table 1:** Characteristics of participants and psychological distress (*N* = 566).

	*N*	Psychological distress					
		HADS ≥ 11		HADS < 10	
		*N*	(%)	*N*	(%)
All participants	566	385	68.0	181	32.0
Age^*^ (mean, SD)	Over all				
	41.9, 10.5	41.3, 10.9		43.1, 9.6	
**Sex**							
Male	205	137	66.8	68	33.2
Female	354	242	68.4	112	31.6
**Marital status**							
Single/divorced	209	151	72.3	58	27.7
Married	350	227	64.9	123	35.1
**Have children under 18 years old**							
Yes	222	144	64.9	78	35.1
None	339	236	69.6	103	30.4
**Work location** ^*^							
NCCH	334	216	64.7	118	35.3
NCCHE	232	169	72.8	63	27.2
**Occupation** ^*^							
Doctor/Dentist	80	40	50.0	40	50.0
Nurse	89	75	84.3	14	15.7
Allied health professionals	114	88	77.2	26	22.8
Office worker/Engineer	129	86	66.7	43	33.3
Researcher/Research assistant	154	96	62.3	58	37.7
**Occupational status**							
Full time	372	264	71.0	108	29.0
Part time	185	116	62.7	69	37.3
Moral distress (mean, SD)	Over all				
	3.44, 2.57	3.42, 2.60		3.47,2.51	
Resilience^*^ (mean, SD)	Over all				
	21.00, 7.45	19.03, 7.13		25.18, 6.31	

HADS, Hospital Anxiety and Depression Scale; NCCH, National Cancer Center Hospital; NCCHE, National Cancer Center Hospital East.

* *p* < 0.05 in univariate analysis.

#### Sample size

We planned to perform a multiple regression analysis to examine the factors related to psychological distress, and we calculated that 10 times as many subjects as the number of independent variables would be required (Peduzzi et al., 1996). Considering the missing data, a minimum of 100 subjects was required. Since NCCH and NCCHE have more than 3,000 medical personnel in total, the estimated sample size was 600 even with a 20% participation rate (Haresaku et al., 2020).

### Statistical analysis

First, the prevalence of psychological distress was assessed. Second, univariate analyses such as an unpaired *t*-test, a chi-square test and a logistic regression analysis were performed as appropriate to identify potential sociodemographic factors associated with psychological distress. Third, a 3-step hierarchical logistic regression model was used to comprehend the degree of association between moral distress, resilience and psychological distress after controlling for potential confounders. Data was analyzed with the SPSS version 26.0 (IBM). All the tests were two-tailed, with a *p* < 0.05.

## Results

Of 3,900 healthcare workers (2,632 women, 67.5%), 566 (14.5%) responded to the survey including 80 doctors/dentists, 89 nurses, 114 allied health professionals, 154 researchers/research assistants, and 129 office workers/engineers. The median age of participants was 43 years (range, 21–68 years) and of whom 354 were female (62.5%). Table 1 presents sociodemographic characteristics of the respondents.

### Prevalence of psychological distress and associated factors

Sixty-eight percent (385/566) responders were above the HADS cutoff. The factors significantly associated with psychological distress were age (*p* = 0.029), work location (*p* = 0.044), and occupation (*p* < 0.001) in univariate analysis (Table 1).

Table 2 shows the results of the hierarchical logistic regression analyses. The first block (sociodemographic and occupational factors) was significant according to the model chi-square statistics. Nurses, allied health professionals and office workers/engineers were significantly associated with higher prevalence of psychological distress (OR = 5.89; 95% CI = 2.56–13.54; *p* < 0.001, OR = 3.39; 95% CI = 1.76–6.61; *p* < 0.001, OR = 2.22; 95% CI = 1.18–4.20; *p* < 0.05). Nurses, allied health professionals and office workers/engineers had a higher prevalence of psychological distress compared to doctors/dentists. The findings from block 2 also revealed a significant model where occupation (nurses, allied health professionals, and office workers/engineers) remained a significant factor. Moral distress was not significantly associated with the prevalence of psychological distress. The model chi-square statistics of blocks 1 and 2 were similar (χ^2^ = 33.06; *p* < 0.001, χ^2^ = 33.35; *p* < 0.001). The third block (sociodemographic and occupational factors, moral distress, resilience) was also significant according to the model chi-square statistics (χ^2^ = 110.06; *p* < 0.001). It suggested the model was a better fit. Nurses, allied health professionals and office workers/engineers as well as HCPs with low resilience were at risk of psychological distress.

**Table 2:** Hierarchical regression model of factors associated with psychological distress.

	Block 1			Block 2			Block 3		
		95% CI			95% CI			95% CI		
	OR	Lower	Upper	OR	Lower	Upper	OR	Lower	Upper
**Age**	0.99	0.97	1.01	0.99	0.98	1.02	1.01	0.99	1.03
**Sex**									
Male	1.00			1.00			1.00		
Female	0.73	0.47	1.13	0.74	0.48	1.14	0.68	0.42	1.09
**Marital status**									
Single/divorced	1.00			1.00			1.00		
Married	0.94	0.57	1.54	0.93	0.57	1.53	0.83	0.49	1.43
**Have children under 18 years old**									
Yes	1.00			1.00			1.00		
None	1.02	0.66	1.59	1.03	0.66	1.59	0.86	0.54	1.39
**Work location**									
NCCHE	1.00			1.00			1.00		
NCCH	0.69	0.47	1.01	0.85	0.56	1.27	0.98	0.63	1.51
**Occupation**									
Doctor/Dentist	1.00			1.00			1.00		
Nurse	5.89^a^	2.56	13.54	5.88^a^	2.56	13.53	4.63^a^	1.90	11.29
Allied health professionals	3.39^a^	1.76	6.61	3.39^a^	1.76	6.61	3.88^a^	1.88	8.00
Office worker/Engineer	2.22^b^	1.18	4.20	2.24^b^	1.18	4.23	2.10^b^	1.06	4.18
Researcher/Research assistant	1.76	0.97	3.21	1.78	0.98	3.25	1.77	0.92	3.39
**Moral distress**				0.98	0.91	1.06	0.98	0.91	1.06
**Resilience**							0.88^a^	0.85	0.91	
**Model statistics**									
χ2	33.06			33.35			110.06		
df	9			10			11		
Model significance	<0.001			<0.001			<0.001		

OR, odds ratio; CI, confidence interval; NCCH, National Cancer Center Hospital; NCCHE, National Cancer Center Hospital East.

a *p* < 0.001.

b *p* < 0.05.

## Discussion

Our study showed that more than two-thirds of HCPs experienced significant psychological distress in oncology hospitals during the first surge of the pandemic. We observed that nurses, allied health professionals, office workers/engineers and HCPs with low resilience were at risk of psychological distress in the final model of the hierarchical logistic regression analyses. Although we hypothesized that HCPs with high moral distress would have psychological distress, moral distress was not significantly associated with the prevalence of psychological distress.

According to a systematic review (Papa et al., 2020), the pooled prevalence of depression and anxiety in healthcare workers was 22.8% and 23.2%, respectively, and our results showed a higher prevalence. Nurses, allied health professionals and office workers/engineers had a higher prevalence of psychological distress compared to doctors/dentists. The high risk of infection could increase the burden on frontline HCPs, especially nurses and allied health professionals including laboratory technicians, pharmacists, radiologists, psychologists, and nutritionists. Matsuo et al. also reported that burnout prevalence was significantly higher among nurses, laboratory medical technologists, radiological technologists, and pharmacists than physicians during the pandemic (Matsuo et al., 2020). Previous studies reported that nurses had more mental health problems than doctors during the COVID-19 pandemic (Lai et al., 2020; Shechter et al., 2020). This may be because nurses spend more time delivering direct patient care.

Although we hypothesized that HCPs with high moral distress would have psychological distress and resilience would be a protective predisposition to psychological distress, moral distress was not significantly associated with the prevalence of psychological distress. This result is not consistent with prior studies (Norman et al., 2021; Petrisor et al., 2021; Schneider et al., 2021; Lake et al., 2022). While providing cancer care under the pandemic, many HCPs were in moderate moral distress. Since there was no association between having moral distress and psychological distress, but there was an association between low resilience and psychological distress, if resilience is considered to be a predisposition that individuals originally have, intervention for medical personnel with low resilience, especially nurses and allied health professionals, should be prioritized. A systematic review showed that psychological interventions to promote resilience in HCPs improved resilience and reduced symptoms of stress and depression (Kunzler et al., 2020). Since there are factors that can be modifiable, interventions on these factors might alleviate COVID-19 related psychological distress in HCPs.

### Limitations

This study has some limitations. First, psychological assessment was performed by a self-reported online survey. Second, the study was conducted in NCCH and NCCHE, and the response rate was low. Findings of this study might not be able to be generalizable. Third, it is not clear whether the results of the investigation conducted in July 2020 will be the same as those conducted during other surges. Finally, the cross-sectional design provides no information on causal relationships.

### Conclusion

During the first surge of the pandemic, a high prevalence of psychological distress was demonstrated among cancer center HCPs. Organizations are recommended to help healthcare workers maintain their mental health and well-being during the pandemic (Walton et al., 2020). At our facility, we use posters to inform about mental health issues among medical personnel and the support system within the hospitals and recommend counseling and psychiatric consultation for those who need it. The study suggests that mental health care should be available to cancer care providers. Since the COVID-19 pandemic is still going on, in addition to the efforts by our facilities, we should consider interventions that promote resilience and conduct a feasibility study of these interventions.

## Data availability statement

The raw data supporting the conclusions of this article will be made available by the authors, without undue reservation.

## Ethics statement

The studies involving human participants were reviewed and approved by the Institutional Review Board and Ethics Committee of National Cancer Center, Japan. An informed consent letter was emailed to participants, and completion of the questionnaire implied their consent.

## Author contributions

MF, YU, and TM designed the study. MO, SG, KO, and MF analyzed the data. MO, MF, SG, NB, RN, TS, and YU interpreted the data. MO and MF were major contributors in writing the manuscript. All authors contributed to the article and approved the submitted version.

## Funding

This study was supported by the research and development grant from National Cancer Center [grant number 30−A-21, 2021-A-23].

## Conflict of interest

The authors declare that the research was conducted in the absence of any commercial or financial relationships that could be construed as a potential conflict of interest.

## Publisher’s note

All claims expressed in this article are solely those of the authors and do not necessarily represent those of their affiliated organizations, or those of the publisher, the editors and the reviewers. Any product that may be evaluated in this article, or claim that may be made by its manufacturer, is not guaranteed or endorsed by the publisher.

## Abbreviations

CD-RISC: The Connor-Davidson Resilience Scale
COVID-19: Coronavirus disease 2019
HADS: The Hospital Anxiety and Depression Scale
HCP: Healthcare provider
NCCH: National Cancer Center Hospital
NCCHE: National Cancer Center Hospital East
PTSD: Post-traumatic stress disorder
